# The Incremental Prognostic Value of Cardiac Computed Tomography in Comparison with Single-Photon Emission Computed Tomography in Patients with Suspected Coronary Artery Disease

**DOI:** 10.1371/journal.pone.0160188

**Published:** 2016-08-03

**Authors:** Heesun Lee, Yeonyee E. Yoon, Jun-Bean Park, Hack-Lyoung Kim, Hyo Eun Park, Seung-Pyo Lee, Hyung-Kwan Kim, Su-Yeon Choi, Yong-Jin Kim, Goo-Yeong Cho, Joo-Hee Zo, Dae-Won Sohn

**Affiliations:** 1 Department of Internal Medicine, Seoul National University College of Medicine, Seoul, Korea; 2 Healthcare System Gangnam Center, Seoul National University Hospital, Seoul, Korea; 3 Department of Cardiology, Cardiovascular Center, Seoul National University Bundang Hospital, Seongnam, Korea; 4 Cardiovascular Center, Seoul National University Hospital, Seoul, Korea; 5 Division of Cardiology, Seoul National University Boramae Medical Center, Seoul, Korea; Nagoya University, JAPAN

## Abstract

**Background:**

Coronary computed tomographic angiography (CCTA) facilitates comprehensive evaluation of coronary artery disease (CAD), including plaque characterization, and can provide additive diagnostic value to single-photon emission computed tomography (SPECT). However, data regarding the incremental prognostic value of CCTA to SPECT remain sparse. We evaluated the independent and incremental prognostic value of CCTA, as compared with clinical risk factors and SPECT.

**Materials and methods:**

A total of 1,077 patients with suspected CAD who underwent both SPECT and cardiac CT between 2004 and 2012 were enrolled retrospectively. Presence of reversible or fixed perfusion defect (PD) and summed stress score were evaluated on SPECT. Presence, extent of coronary atherosclerosis and diameter stenosis (DS) were evaluated on CCTA. Plaque composition was categorized as non-calcified, mixed, or calcified according to the volume of calcified component (>130 Hounsfield Units). Patients were followed up for the occurrence of adverse cardiac events including cardiac death, non-fatal myocardial infarction, unstable angina, and late revascularization (>90 days after imaging studies).

**Results:**

During follow-up (median 23 months), adverse cardiac events were observed in 71 patients (6.6%). When adjusted for clinical risk factors and SPECT findings, the presence of any coronary plaque, any plaque in ≥3 segments, coronary artery calcium score (CACS) ≥400, a plaque ≥50% DS, presence of non-calcified plaque (NCP) or mixed plaque (MP), and NCP/MP in ≥2 segments were independent predictors of adverse cardiac events; however, the presence of calcified plaque (CP) was not. Conventional CCTA findings, including CACS ≥400 and a plaque ≥50% DS, demonstrated incremental prognostic value over clinical risk factors and SPECT (χ² 54.19 to 101.03; *p* <0.001). Addition of NCP/MP in ≥2 segments resulted in further significantly improved prediction (χ² 101.03 to 113.29; *p* <0.001).

**Conclusion:**

Comprehensive CCTA evaluation of coronary atherosclerosis provides independent and incremental prognostic value in relation to SPECT evaluation of myocardial ischemia. Specifically, segmentally-analyzed plaque composition with CCTA provides further risk stratification in addition to CACS and DS.

## Introduction

When evaluating patients with suspected coronary artery disease (CAD), noninvasive imaging has become increasingly used for risk stratification and decisions regarding further management [1, 2]. Traditionally, myocardial perfusion imaging with single-photon emission computed tomography (SPECT) has been widely used for the diagnosis of CAD, based on the visualization of inducible ischemia [3, 4]. More recently, cardiac computed tomography (CT) has emerged as a noninvasive modality for evaluating CAD; the coronary artery calcium score (CACS) estimates the coronary atherosclerotic burden, and coronary computed tomographic angiography (CCTA) allows direct visualization of CAD [3–5]. Because CCTA identifies coronary atherosclerosis rather than myocardial ischemia, previous studies have addressed the association between CCTA and SPECT in the evaluation of CAD [3, 6]. While several comparative studies have demonstrated that CCTA can provide additive diagnostic value to SPECT, data regarding the incremental prognostic value of CCTA are scarce [4, 7–9]. Moreover, it remains poorly understood how comprehensive coronary plaque evaluation using CCTA can improve risk stratification in SPECT. Specifically, while CCTA has a unique ability to provide information on plaque composition, it is not well known whether plaque composition can further enhance risk stratification. Therefore, in this study, we aimed to evaluate whether CCTA can provide independent and incremental prognostic value as compared with SPECT in patients with suspected CAD. Furthermore, we tried to determine whether analysis of plaque composition by CCTA provides incremental prognostic value over clinical risk factors, SPECT, and conventional CCTA findings including CACS and stenosis severity.

## Materials and Methods

### Study population

**Fig 1:**
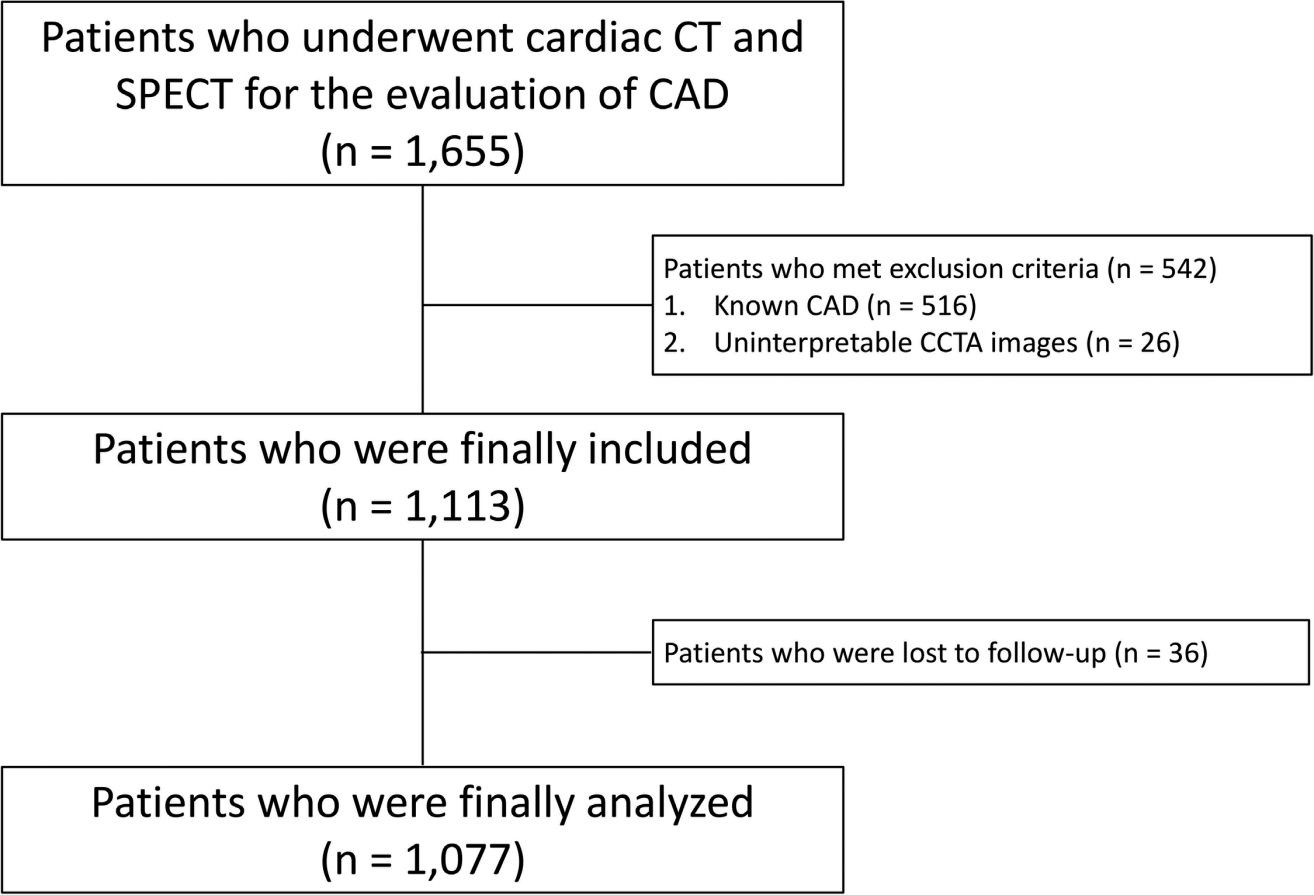
CONSORT diagram of the study population selection. CCTA, coronary computed tomographic angiography; SPECT, single-photon emission computed tomography; CAD, coronary artery disease.

We retrospectively reviewed the medical records of 1,655 consecutive adult patients who underwent both cardiac CT and SPECT within 90 days for the evaluation of CAD at Seoul National University Hospital and Seoul National University Bundang Hospital between 2004 and 2012. The cardiac CT protocols included both calcium scan and CCTA [10, 11]. Among this initial cohort, we excluded 516 patients with known CAD (stable angina or acute coronary syndrome with or without coronary revascularization) and 26 patients with uninterpretable imaging data. Therefore, 1,113 were eligible for inclusion in our study. However, 36 patients (3.2%) were lost to follow-up, resulting in 1,077 patients (96.8%) finally being analyzed analysis. (Fig 1) The study protocol conforms to the guidelines in the Declaration of Helsinki. The Institutional Review Board of Seoul National University Hospital and Seoul National University Bundang Hospital approved this retrospective study, and waived the requirement for written informed consent.

### SPECT image acquisition and analysis

Myocardial SPECT (CardioMD; ADAC Vertex V60, Philips Medical Systems Inc, Cleveland, OH, USA) was performed using technetium-99m tetrofosmin or sestamibi as the radiotracer. Rest images were acquired 1 h after intravenous administration of 8–10 mCi technetium-99 m. Then, stress images were obtained at peak stress in sequence with a continuous infusion of adenosine (0.14 mg/kg/min) or dipyridamole (0.142 mg/kg/min), and subsequent radiotracer (25 mCi technetium-99 m) injection 3 minutes after beginning stress. A dual-headed gamma camera was used with a circular or elliptical 180-degree acquisition for 64 projections at 25 sec/projection. Gated images were obtained 90 minutes after stress from 3 planes of the left ventricle: the short axis, long vertical axis, and long horizontal axis with electrocardiographic (ECG) synchronization [10, 12].

SPECT images were interpreted by consensus of two nuclear physicians, with 23 and 17 years’ experience, blinded to clinical information, according to the recommendations of the American Society of Nuclear Cardiology, using a 17-segment model [13]. Patients were categorized according to the presence or absence of perfusion defect (PD), as determined on the stress image (segmental tracer activity <75% of maximum), and divided into fixed (irreversible change on the resting phase) and reversible (≥10% increase in tracer uptake on the resting phase) PD [14]. A summed stress score (SSS) was calculated, as previously described, and classified according to SSS ≤4 or >4, which has been established as a reliable marker of significant myocardial ischemia [15].

### Cardiac CT image acquisition and analysis

Cardiac CT was performed using a 64-detector row CT scanner (SOMATOM Definition, Siemens Medical Solutions, Forchheim, Germany; Brilliance 64, Philips Medical Systems, Best, the Netherlands) with a 64 × 0.625 mm slice collimation, rotation time of 0.42 sec, tube voltage of 120 kV, and a tube current of 150 mAs for calcium scoring and 800 mAs for CCTA. Sublingual nitroglycerin was administered immediately before scanning. In patients with a heart rate ≥70 beats/min, a ß-blocker (metoprolol or esmolol) was administered to stabilize the heart rate. CCTA images were acquired in a craniocaudal direction with retrospective ECG-gating and dose modulation. A bolus of 60–80 mL of ionized contrast (IOMERON 400, Bracco Imaging SpA, Milan, Italy) was injected at 4 mL/s, followed by a 50 mL saline flush. Unenhanced CT and contrast-enhanced CT angiograms were reconstructed with a non-overlapping slice thickness of 3 and 0.6 mm, respectively [10].

CCTA images were transferred to an offline 3-dimensional workstation, and independently analyzed by two trained radiologists blinded to clinical information. The presence, severity, and composition of coronary atherosclerotic plaques were evaluated with a per-segment analysis, according to the modified American Heart Association 15-segment criteria [16, 17]. A coronary atherosclerotic plaque was defined as any clearly discernible lesion >1 mm^2^ that could be discriminated from the coronary artery in at least 2 independent image planes. Patients were categorized as having no plaque, plaque <50% DS, and ≥50% DS. Plaque composition was classified as non-calcified (<30% calcified plaque volume), mixed (30% to 70%), or calcified (>70%), according to the calcified component (>130 Hounsfield Units [HU]) [18, 19]. The CACS was measured using the Agatston scoring system, as previously described [20], and graded in the following manner: 0, 1–399, and ≥400 [10].

### Clinical follow-up

Follow-up information was obtained via review of medical records and telephone interviews performed by the patient’s clinical physician. The primary outcome was the occurrence of major adverse cardiac events, including cardiac death, non-fatal myocardial infarction (MI), unstable angina (UA), and late revascularization. Cardiac death, non-fatal MI, and UA were defined according to the Third Universal Definition of MI, American College of Cardiology Foundation, and the American Heart Association [17, 21]. Late revascularization was defined as any revascularization, either by percutaneous coronary intervention or coronary artery bypass graft, after 90 days from the last SPECT or cardiac CT examination. Coronary revascularization within 90 days of the most recent imaging test was excluded as censoring, to subtract any examination-driven revascularizations from clinical events.

### Statistical analysis

Data were presented as numbers and percentages for categorical variables, and mean ± standard deviation (SD) for continuous variables. Differences between continuous variables were compared using Student’s *t* test for independent samples, while those between categorical variables were analyzed using the χ^2^ test or Fisher’s exact test, as appropriate. To identify a criterion of involved segment number for the prediction of further clinical events, receiver-operating characteristic (ROC) curves were plotted, and the optimal cut-off values were determined according to the maximum sum of sensitivity and specificity. The annual event rate was compared in accordance with results of cardiac CT and SPECT. Kaplan-Meier survival curves were drawn to estimate the risk of adverse cardiac events during follow-up in the entire cohort, and in subgroups according to PD on SPECT, with comparison performed using log-rank tests. The Cox proportional hazard model with a forward selection method was used to estimate the risk of adverse cardiac events, according to clinical and imaging parameters. The risk of adverse cardiac events was expressed as a hazard ratio (HR) and corresponding 95% confidence interval (CI) from univariate and multivariate analyses in order. Additionally, to evaluate the incremental prognostic value of plaque composition over clinical risk factors, SPECT, and conventional CT findings (including CACS) in predicting cardiac events, a sequential Cox analysis using 3 incremental models was performed. Model 1 consisted of clinical risk factors represented by the Framingham Risk Score (FRS) + PD on SPECT; model 2 as FRS + PD on SPECT + CACS ≥400 + DS ≥50%; and model 3 as FRS + PD on SPECT + CACS ≥400 + DS ≥50% + non-calcified or mixed plaque in ≥2 segments on cardiac CT. The change in overall log-likelihood ratio χ^2^ was used to assess increases in predictive power with subsequent parameters. In ROC analysis for the 3 models, area under the curve (AUC) for the prediction of further clinical events was used to evaluate the incremental prognostic value of the combined approach; the difference between AUCs was tested for significance [22]. A value of *p* <0.05 was considered statistically significant. All analyses were performed using SPSS 22.0 (IBM, Chicago, Illinois, USA) and MedCalc for Windows, version 13.1.2.0 (MedCalc Software, Ostend, Belgium).

## Results

The detailed clinical characteristics and imaging results of the 1,077 patients (mean age, 62 ± 10 years, 63.2% male) included in the final analysis are summarized in Table 1. On the basis of the FRS, 32% of the studied patients were classified as low risk (10-year risk <10%), 45% as moderate risk (10–20%), and 23% as high risk (>20%). Primary indications for cardiac CT and SPECT were chest pain (56.1%) and dyspnea (17.8%). Asymptomatic patients (138, 12.8%) primarily consisted of those with multiple cardiac risk factors and those with planned non-coronary cardiac surgery. The average CACS was 258; a CACS ≥400 was observed in 183 patients (17.0%). Coronary atherosclerotic plaque on CCTA was observed in 797 patients (74.0%); 490 (45.5%) had a significant CAD with plaque ≥50% DS. Non-calcified plaques (NCP) were observed in 434 patients (40.3%), mixed plaques (MP) in 350 (32.5%), and calcified plaques (CP) in 503 patients (46.7%). Any type of PD on SPECT was observed in 328 of 1,077 patients (30.5%); 63 (5.8%) had a fixed PD, and 170 (15.8%) exhibited an SSS ≥4.

**Table 1:** Baseline clinical and imaging characteristics.

Variables	Total patients (n = 1,077)	
**Clinical parameters**		
Age, years	62 ± 10	
Male, %	681 (63.2)	
Smoking, %	418 (38.9)	
Body mass index, kg/m^2^	25 ± 3	
Hypertension, %	453 (42.1)	
Diabetes mellitus, %	592 (55.0)	
Dyslipidemia, %	485 (45.3)	
Framingham Risk Score, %	15 ± 9	
Low	349 (32.4)	
Intermediate	479 (44.5)	
High	249 (23.1)	
**Indications for tests**		
Chest pain, %	605 (56.1)	
Non-anginal chest pain, %	165 (15.3)	
Atypical angina, %	180 (16.7)	
Typical angina, %	260 (24.1)	
Dyspnea, %	192 (17.8)	
Palpitations, %	31 (2.9)	
Presyncope/syncope, %	15 (1.4)	
Others, %	96 (8.9)	
Asymptomatic, %	138 (12.8)	
**Medications at baseline**		
Antiplatelet agents, %	332 (30.8)	
RAS blocker, %	195 (18.1)	
Beta blocker, %	91 (8.4)	
Long–acting nitrate, %	44 (4.1)	
Statin, %	170 (15.8)	
**Medications at 3 months after last exam of SPECT and cardiac CT**		
Antiplatelet agents, %	687 (63.8)	
RAS blocker, %	381 (35.4)	
Beta blocker, %	212 (19.7)	
Long–acting nitrate, %	67 (6.2))	
Statin, %	469 (43.5)	
**Myocardial SPECT findings**		
Presence of any PD	328 (30.5)	
Presence of fixed PD	63 (5.8)	
SSS ≥4	170 (15.8)	
**Cardiac CT findings**		
CACS		
0	338 (31.4)	
1–399	556 (51.6)	
≥400	183 (17.0)	
Coronary CT angiography		
Presence of any plaque	797 (74.0)	
Presence of plaque ≥50% DS	490 (45.5)	
Presence of NCP	434 (40.3)	
Presence of MP	350 (32.5)	
Presence of CP	503 (46.7)	

RAS = renin-aldosterone system; SPECT = single-photon emission computed tomography; CT = computed tomography; CACS = coronary artery calcium score; DS = diameter stenosis; NCP = non-calcified plaque; MP = mixed plaque; CP = calcified plaque; PD = perfusion defect; SSS = summed stress scores.

During a median follow-up duration of 23 months (interquartile range 10 to 39 months), 71 patients (6.6%) experienced adverse cardiac events, including 2 patients with cardiac death, 7 with non-fatal MI, 23 with unstable angina, and 39 with late revascularization (12 coronary artery bypass grafts and 27 percutaneous coronary interventions). When stratified by SPECT findings, presence of any PD was associated with increased risk (annual event rate 1.2% vs. 4.2%; *p* <0.001). In patients with any PD, SSS ≥4 was associated with a further increase in risk (annual event rate 3.3% vs. 4.9%; *p* = 0.008), while the presence of fixed PD was not (3.9% vs. 3.0%; *p* = 0.252). When stratified by cardiac CT results, the occurrence of adverse cardiac events increased significantly with increasing CACS (annual event rate 0.6% vs. 1.8% vs. 6.1%; *p* <0.001) and with presence of coronary atherosclerotic plaque (0.3% vs. 2.8%; *p* <0.001) (Table 2). In patients with coronary plaque, plaques ≥50% DS were associated with further increased risk (annual event rate 0.9% vs. 4.0%; *p* <0.001). While the presence of CP did not exhibit a significant association with further cardiac events (annual event rate 2.6% vs. 2.5%; *p* = 0.886), both NCP and MP were associated with further risk of adverse cardiac events (1.4% vs. 3.2%; *p* = 0.002, and 1.4% vs. 4.6%; *p* <0.001, respectively).

**Table 2:** The occurrence of adverse cardiac event according to cardiac CT and myocardial SPECT.

Variables	Adverse cardiac event	Annual event rate (%)	Log-rank *p* value
	No./No. at risk (%)		
**Myocardial SPECT findings**			
In the entire cohort (n = 1,077)			
Absence of any PD	25/749 (3.3)	1.2	<0.001
Presence of any PD	46/328 (14.0)	4.2	
In patients with any PD (n = 329)			
Absence of fixed PD	40/265 (15.1)	3.9	0.252
Presence of fixed PD	6/63 (9.5)	3.0	
SSS <4	14/159 (8.8)	3.3	0.008
SSS ≥4	32/169 (18.9)	4.9	
**Cardiac CT findings**			
In the entire cohort (n = 1,077)			
CACS 0	6/338 (1.8)	0.6	<0.001
CACS 1–399	35/556 (6.3)	1.8	
CACS ≥400	30/183 (16.4)	6.1	
Absence of plaque	2/280 (0.7)	0.3	<0.001
Presence of plaque	69/797 (8.7)	2.8	
In patients with plaque (n = 797)			
Presence of plaque of <50% DS	9/307 (2.9)	0.9	<0.001
Presence of plaque of ≥50% DS	60/490 (12.2)	4.0	
Absence of NCP	24/363 (4.4)	1.4	0.002
Presence of NCP	47/434 (9.9)	3.2	
Absence of MP	20/447 (4.5)	1.4	<0.001
Presence of MP	51/350 (14.6)	4.6	
Absence of CP	26/294 (8.8)	2.6	0.886
Presence of CP	43/503 (8.5)	2.5	
Any plaque in <3 segments	16/342 (4.7)	1.4	0.001
Any plaque in ≥3 segments	53/455 (11.6)	3.9	
NCP/MP in <2 segments	19/410 (4.6)	1.4	<0.001
NCP/MP in ≥2 segments	50/387 (12.9)	4.1	

No = numbers; other abbreviations are same as Table 1.

**Fig 2:**
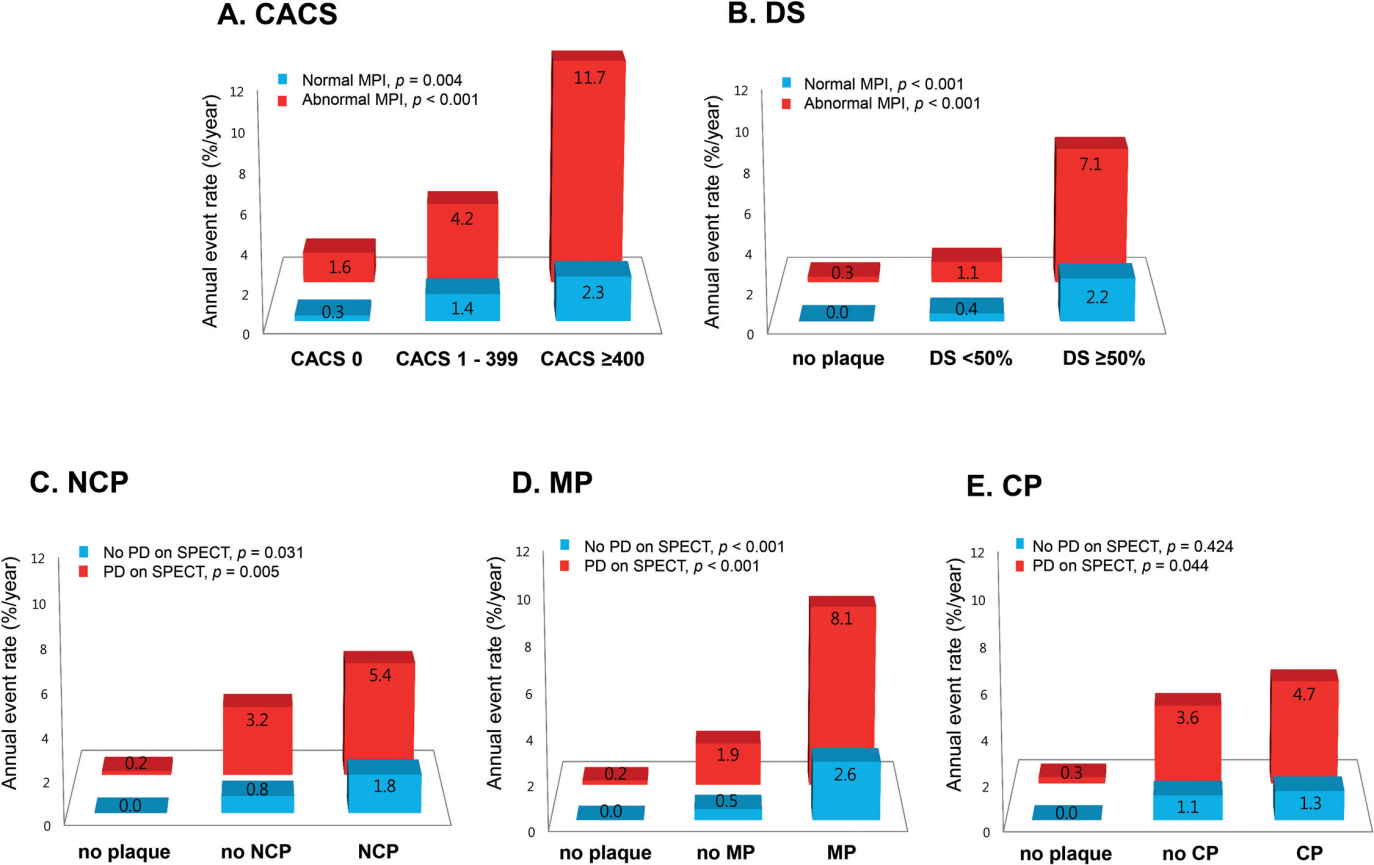
Improved prediction of adverse cardiac events with integration of cardiac computed tomography (CT) and single-photon emission computed tomography (SPECT). Comparison of the annual event rate according to (A) increasing coronary artery calcium score (CACS), (B) worsening diameter stenosis (DS), (C) presence of non-calcified plaque (NCP), (D) mixed plaque (MP), and (E) calcified plaque (CP) between patients with and without perfusion defect (PD) on SPECT.

**Fig 3:**
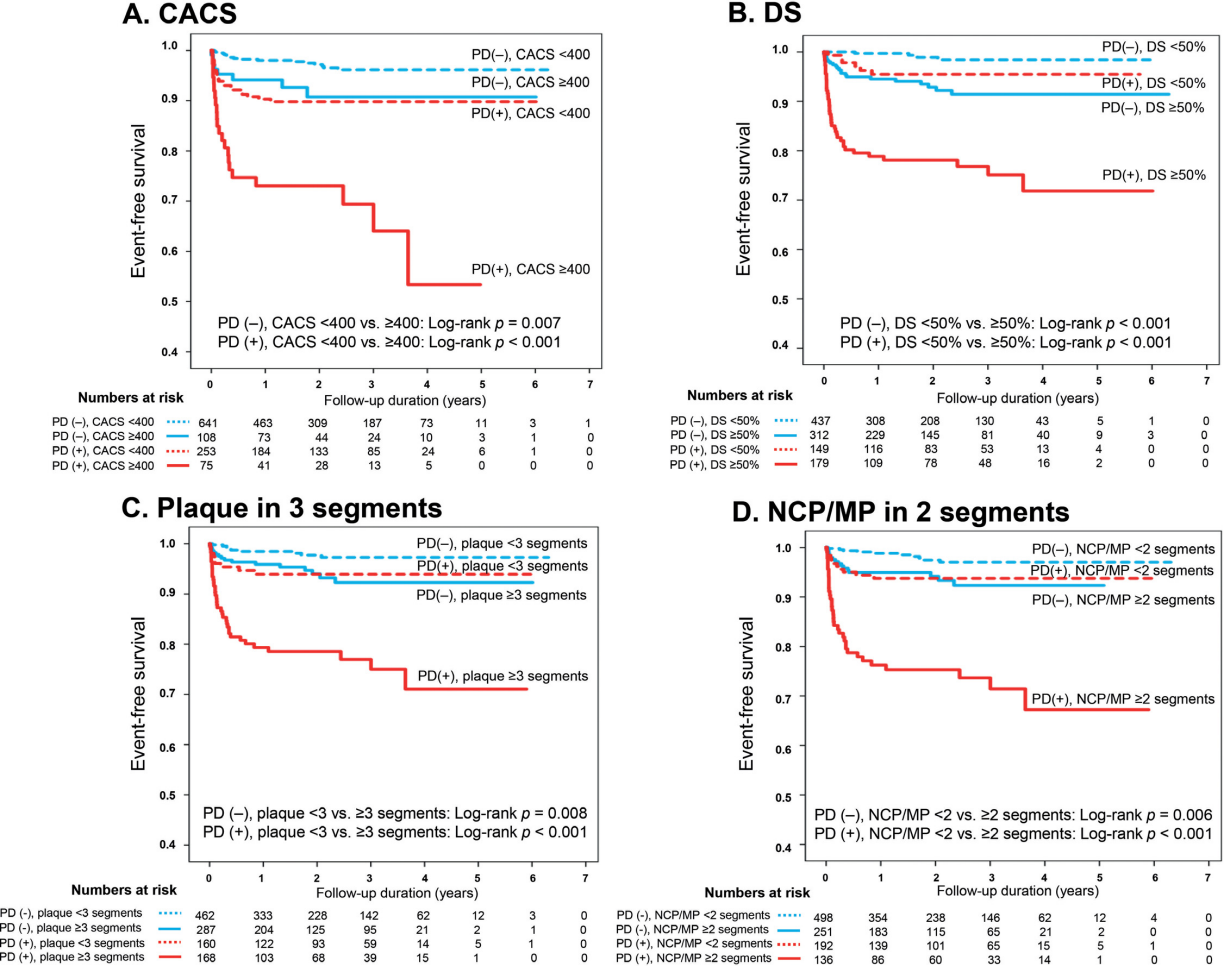
Kaplan-Meier survival curves for adverse cardiac events from combined cardiac CT and SPECT information. Event-free survival curves stratified by (A) coronary artery calcium score (CACS) of 400 and presence or absence of perfusion defect (PD), (B) diameter stenosis (DS) of 50% and presence or absence of PD, (C) presence of plaque in 3 or more segments of the coronary tree and presence or absence of PD, and (D) presence of non-calcified plaque (NCP) or mixed plaque (MP) in 2 or more segments of the coronary tree and presence or absence of PD.

Integrating cardiac CT and SPECT improved prediction of adverse cardiac events (Fig 2). In both groups with and without PD on SPECT, the annual event rate for adverse cardiac events increased significantly with increasing CACS (*p* <0.001, and *p* = 0.004, respectively) and with worsening DS (*p* <0.001 for both) (Fig 2A and 2B). The presence of NCP was associated with an increased annual event rate in groups with and without PD on SPECT (*p* = 0.005, and *p* = 0.031, respectively) (Fig 2C). The presence of MP also resulted in a significantly increased annual event rate in both groups (*p* <0.001 for both) (Fig 2D). However, CP was not associated with an increased annual event rate in the group without PD (*p* = 0.424), and showed a weak association with the annual event rate in the group with PD (*p* = 0.044) (Fig 2E). In ROC analysis, the optimal cut-off for the number of involved segments was determined as 3 in the total plaque count and 2 in NCP/MP (S1 Fig). Kaplan-Meier survival curve analyses demonstrated that CACS ≥400, plaque DS ≥50%, plaque in ≥3 segments, and NCP/MP in ≥2 segments improved risk stratification beyond that achieved when using only the presence or absence of PD on SPECT (Fig 3).

Cox regression analyses of clinical characteristics and cardiac CT and SPECT findings for adverse cardiac events are summarized in Table 3. Among clinical variables, age, male sex, FRS, and the use of antiplatelet agents, nitrate, and statins at 3 months after the last imaging study were strongly associated with adverse cardiac events. The presence of any PD, SSS ≥4 on SPECT, CACS ≥400, presence of any atherosclerotic plaque, and presence of plaque ≥50% DS on cardiac CT were significant predictors for adverse cardiac events. The presence of NCP/MP showed a >7-fold hazard increase for adverse cardiac events, while the presence of CP did an about 2-fold increase (unadjusted HR 7.35; 95% CI 3.18–16.97; *p* <0.001, and unadjusted HR, 1.83; 95% CI 1.13–2.97; *p* = 0.013, respectively). Presence of any plaque in ≥3 segments and NCP or MP in ≥2 segments was associated with an approximately 4-fold hazard increase for adverse cardiac events (unadjusted HR 4.11; 95% CI 2.40–7.03; p = 0.001, and unadjusted HR 4.24; 95% CI, 2.54–7.07; p <0.001). In multivariate analysis (adjusted for FRS, the use of antiplatelet agents, nitrate, and statins at 3 months after the last imaging study, and presence of PD on SPECT), CACS ≥400, the presence of any coronary plaque, any plaque in ≥3 segments, plaque of ≥50% DS, presence of NCP/MP, and NCP/MP in ≥2 segments maintained independent associations with the risk of adverse cardiac events, while the presence of CP did not (Table 3).

**Table 3:** Univariate and multivariate analysis of factors associated with adverse cardiac event.

	Univariate analysis		Multivariate analysis^†^	
Variables	Unadjusted HR (95% CI)	*p* value	Adjusted HR (95% CI)	*p* value
**Clinical variables**				
Age	1.03 (1.00–1.05)	0.037	-	-
Male	2.13 (1.20–3.77)	0.009	-	-
Smoking	1.36 (0.83–2.22)	0.218	-	-
Hypertension	1.58 (0.99–2.54)	0.057	-	-
Diabetes mellitus	1.15 (0.71–1.85)	0.568	-	-
Dyslipidemia	0.86 (0.54–1.38)	0.535	-	-
FRS	1.05 (1.02–1.07)	<0.001	-	-
Antiplatelet agents*	3.70 (1.77–7.74)	<0.001	-	-
RAS blocker *	1.17 (0.72–1.87)	0.530	-	-
Beta blocker*	1.22 (0.71–2.12)	0.468	-	-
Long–acting nitrate*	2.82 (1.48–5.36)	0.002	-	-
Statin*	1.77 (1.09–2.85)	0.020	-	-
**Myocardial SPECT findings**				
Presence of any PD	4.19 (2.57–6.83)	<0.001	-	-
Presence of fixed PD	1.41 (0.61–3.27)	0.418	-	-
SSS ≥4	4.62 (2.88–7.40)	<0.001	-	-
**Cardiac CT findings**				
CACS ≥400	4.04 (2.52–6.50)	<0.001	2.77 (1.71–4.49)	<0.001
Presence of plaque	7.36 (3.77–14.39)	<0.001	7.37 (1.75–31.06)	0.006
Plaque in ≥3 segments	4.11 (2.40–7.03)	0.001	2.65 (1.51–4.64)	0.001
Plaque ≥50% DS	7.36 (3.77–14.39)	<0.001	5.18 (2.58–10.49)	<0.001
Presence NCP/MP	7.35 (3.18–16.97)	<0.001	5.20 (2.21–12.26)	<0.001
NCP/MP in ≥2 segments	4.24 (2.54–7.07)	<0.001	3.02 (1.78–5.14)	<0.001
Presence of CP	1.83 (1.13–2.97)	0.013	1.23 (0.75–2.02)	0.407

* Information about medication in Table 3 was collected after 3 months from the last exam of SPECT and cardiac CT.

† Multivariate analysis was performed by adjusting to FRS, antiplatelet agent, long-acting nitrate, statin after 3 months from the last imaging study and presence of PD on SPECT.

HR = hazard ratio; CI = confidence interval; FRS = Framingham risk score; other abbreviations are same as Tables 1 and 2.

To assess the incremental prognostic value of cardiac CT variables over clinical risk factors and SPECT variables, FRS, presence of PD on SPECT, CACS ≥400, DS ≥50%, and presence of NCP/MP in ≥2 segments were included in the Cox model (Table 4). When conventional cardiac CT variables (CACS ≥400 and the presence of plaque ≥50% DS) were added to the combination of FRS and the presence of PD on SPECT, a statistically significant increase in the global χ^2^ value was observed (54.19 vs. 101.03, *p* <0.001). The addition of presence of NCP/MP in ≥2 segments resulted in further risk stratification (global χ^2^ 101.03 vs. 113.29; *p* <0.001) (S2 Fig). In the ROC curve analysis, the AUC for the combination of FRS and PD on SPECT (AUC 0.74; 95% CI 0.72–0.77) was significantly increased with the addition of CACS ≥400 and plaque ≥50% DS (AUC 0.81; 95% CI 0.79–0.84; *p* = 0.003). The addition of NCP/MP in ≥2 segments significantly increased AUC (AUC 0.85; 95% CI 0.82–0.88; *p* = 0.045) (S3 Fig).

**Table 4:** Incremental prognostic value of cardiac CT findings for adverse cardiac event.

Models	Hazard ratio	*p* value	Global χ^2^	*p* value for comparison (for global χ^2^)	AUC	*p* value for comparison (for AUC)
	(95% CI)				(95% CI)	
**Model 1**			54.19		0.74 (0.72–0.77)	
FRS	1.04 (1.02–1.07)	<0.001				
PD on SPECT	4.02 (2.47–6.57)	<0.001				
**Model 2**			101.03	<0.001	0.81 (0.79–0.84)	0.003
FRS	1.03 (1.01–1.05)	0.014				
PD on SPECT	3.42 (2.09–5.59)	<0.001				
CACS ≥400	1.97 (1.21–3.23)	0.007				
DS ≥50%	4.82 (2.40–9.70)	<0.001				
**Model 3**			113.29	<0.001	0.85 (0.82–0.88)	0.046
FRS	1.03 (1.01–1.05)	0.016				
PD on SPECT	3.27 (2.00–5.36)	<0.001				
CACS ≥400	2.19 (1.34–3.59)	0.002				
DS ≥50%	3.34 (1.62–6.90)	0.001				
NCP/MP in ≥2 segments	2.45 (1.43–4.19)	0.001				

AUC = area under curve; other abbreviations are same as Table 1 and 2.

## Discussion

The main finding of the present study is that comprehensive cardiac CT evaluation of coronary atherosclerosis and its characterization adds significant risk prediction beyond clinical risk factors and SPECT findings in patients with suspected CAD. The presence, extent, and characteristics of coronary atherosclerosis, as well as the presence of significant stenosis of ≥50% luminal narrowing provide independent and incremental prognostic value. Specifically, the presence of NCP or MP in ≥2 segments results in improved risk stratification, even over conventional cardiac CT findings, when added to clinical risk factors and SPECT evaluation of myocardial ischemia.

In patients with suspected CAD, noninvasive imaging tests have been increasingly used for risk stratification and decision-making processes regarding treatment. Traditionally, noninvasive evaluation was based on the functional significance of CAD. SPECT, which is able to visualize inducible ischemia, has played a central role in the risk assessment of patients with suspected CAD [23]. More recently, CCTA has been proposed as an alternative imaging modality for the evaluation of CAD, and has changed the paradigm of risk stratification [3, 4]. Compared to traditional approaches that focus on evaluating myocardial ischemia, CCTA allows direct visualization of coronary atherosclerosis. Although CCTA has limited value for detecting ischemia-causing lesions that require revascularization, the presence of coronary atherosclerosis on CCTA, whether obstructive or non-obstructive, has been associated with increased risk of cardiac events [24–26]. However, to date, there are sparse data regarding the potential for cardiac CT to provide complementary prognostic information when performed with SPECT. To our best knowledge, only 1 study by van Werkhoven et al. has reported that CCTA provides prognostic value to SPECT [4]. In 541 patients with suspected CAD, DS ≥50% improved risk stratification in both groups with and without abnormal SPECT findings, and was shown to provide independent and incremental prognostic value over SPECT [4]; these observations are in accordance with those observed herein. In the current study, we found that high CACS and the presence and extent of any coronary plaque on CCTA also enhanced risk stratification, and were independent predictors even after adjustment for clinical risk factors and SPECT findings. Our results support the role of cardiac CT in CAD risk stratification, and suggest that cardiac CT facilitates further risk stratification in both groups with and without myocardial ischemia.

CCTA permits noninvasive evaluation of coronary atherosclerotic plaques beyond simple luminal narrowing and plaque type (as defined by calcium content) [27]. Thus, high-risk plaque features have been the target of noninvasive imaging with CCTA. An early CCTA study demonstrated the feasibility of detecting high-risk plaques [17], and subsequent studies have shown an association between plaque features, such as large plaque burdens, positive remodeling, spotty calcification, plaque with low HU values, and the napkin-ring sign, with acute coronary syndrome [28–31]. However, the majority of studies have been performed exclusively in patients with acute coronary syndrome, not in patients with stable angina. Although van Werkhoven et al. identified that the presence of NCP had independent and incremental prognostic value over SPECT in patients with suspected CAD [4], they did not examine the prognostic value of MP and CP [4]. Recently, Hou et al. evaluated coronary plaque composition using CCTA in 5,007 outpatients with suspected CAD, and reported that the risk of adverse cardiac events was significantly higher in cases of NCP and MP than CP [32]. However, they did not compare CCTA with functional studies, and were thus unable to evaluate whether determination of plaque composition could enhance risk stratification over a functional test. In concordance with this previous study [32], we have demonstrated that the presence of NCP or MP, rather than CP, has prognostic value in patients with suspected CAD. Moreover, we also found that NCP/MP in ≥2 segments enhances risk stratification in both groups with and without myocardial ischemia. Presence of NCP/MP in ≥2 segments maintained a 3-fold hazard increase for adverse cardiac events when adjusted for clinical risk factors and SPECT findings. These results support the hypothesis that soft, lipid-filled plaques, despite rarely being involved with myocardial ischemia, are more vulnerable leading to subsequent clinical events.

Although there is growing evidence of the prognostic value of CCTA, CCTA has limited value for the detection of ischemia-causing lesions that require revascularization [33, 34]. Because of the inherent limited ability of CCTA to determine the physiological significance of CAD, other noninvasive imaging studies such as SPECT, stress echocardiography, and magnetic resonance imaging are commonly required in daily clinical practice. In that reason, recent efforts have focused on using CCTA to evaluate plaque burden [11], and to detect “vulnerable” plaques for identification of high-risk patients who may benefit from intensive medical treatment [35]. In our study, the presence of NCP/MP in ≥2 segments was associated with incremental value in addition with clinical risk factors, SPECT, CACS, and DS ≥50% for the prediction of adverse cardiac events. These results suggest the necessity of close monitoring and aggressive medical treatment in patients with NCP or MP, even though these lesions do not require revascularization. For sure, since we could not evaluate the efficacy of aggressive medical treatment according to plaque -composition in this observational study, further study is required to observe the effects of this methodology in clinical practice. More recently, technical advances have made the development of myocardial CT perfusion imaging feasible, and the role of cardiac CT is expanding from the evaluation of coronary atherosclerosis to the assessment of functional significance of CAD [8, 9]. Therefore, further studies are required to evaluate whether improving diagnostic accuracy by combining the anatomic aspect of CCTA with physiological assessment via myocardial CT perfusion could reduce the risks associated with invasive procedures and lead to improved patient outcomes. In addition, we also need to evaluate whether determination of plaque composition can provide an incremental prognostic value even when performed with cardiac CT protocols including myocardial CT perfusion imaging.

There are several limitations in this study. First, this study was retrospective in nature. Therapeutic procedures were not guided by a specific protocol, and might have been influenced by cardiac CT and/or SPECT results. However, such effects are inevitable in a study observing the clinical diagnostic and treatment pathway. In addition, coronary revascularizations within 90 days after imaging studies, usually performed as a result of cardiac CT and/or SPECT, were excluded from cardiac events. Moreover, patients were censored for the end point of all cardiac events after the first revascularization, irrespective of timing; thus, differences in event rates were not influenced by complications associated with revascularization. Second, many of our patients were symptomatic, and exhibited more risk factors than the general population. Thus, the generalizability of our results may be limited, and further community-based studies are required. However, to our best knowledge, this is the largest study evaluating the prognostic value of cardiac CT in relation to SPECT findings. Furthermore, we have highlighted the importance of NCP and MP, even in patients without evidence of myocardial ischemia. Finally, the extent of coronary atherosclerosis was evaluated in a semiquantitative manner by identifying the number of involved segments of coronary tree. Although identifying a volumetric measure for coronary plaque burden with CCTA was suggested, accuracy remains limited by insufficient spatial resolution, blooming, and motion artifacts [36]. Instead, we selected cardiac CT parameters that are directly applicable in clinical practice.

## Conclusions

Comprehensive cardiac CT evaluation of coronary atherosclerosis provides independent and incremental prognostic value in relation to SPECT evaluation of myocardial ischemia in patients with suspected CAD. Particularly, presence and extent of NCP/MP, as evaluated by cardiac CT, allows further risk stratification in addition to the CACS and DS.

## Supporting Information

S1 Fig The optimal cut-off value of involved segments to predict adverse cardiac events. In receiver operating characteristics (ROC) analysis, the optimal cut-off for the number of involved segments is determined as 3 in the total plaque count (A) and 2 in non-calcified plaque (NCP)/mixed plaque (MP) (B), respectively.
(JPG)

S2 Fig Incremental prognostic value of cardiac computed tomography (CT) variables by providing global χ^2^ scores. Bar graph illustrates the incremental prognostic value of cardiac CT variables in predicting adverse cardiac events by providing global **χ**^**2**^ scores. The addition of conventional cardiac CT variables (coronary artery calcium score [CACS] ≥400 and plaque ≥50% diameter stenosis [DS]) provides incremental prognostic information to Framingham Risk Score (FRS) and perfusion defect (PD) on single-photon emission computed tomography (SPECT). Furthermore, the addition of presence of non-calcified plaque (NCP)/mixed plaque (MP) in ≥2 segments allows further risk stratification.
(JPG)

S3 Fig Incremental prognostic value of cardiac computed tomography (CT) variables by comparing area under curve (AUC) values. The receiver-operating characteristic (ROC) curves of 3 models depict the incremental prognostic value of cardiac CT variables in predicting adverse cardiac events by comparing AUC values. The AUC increases gradually from 0.74 (1: the Framingham Risk Score [FRS] + perfusion defect [PD] on single-photon emission computed tomography [SPECT]) to 0.81 (2: adding coronary artery calcium score [CACS] ≥400 and plaque ≥50% diameter stenosis [DS] to model 1), and from 0.81 to 0.85(3: adding non-calcified plaque [NCP]/mixed plaque [MP] in ≥2 segments to model 2).
(JPG)
