# Supplementary figures and images for: The Incremental Prognostic Value of Cardiac Computed Tomography in Comparison with Single-Photon Emission Computed Tomography in Patients with Suspected Coronary Artery Disease
Source: PLoS One. 2016 Aug 3;11(8):e0160188. doi: 10.1371/journal.pone.0160188 (PMC4972322; doi:10.1371/journal.pone.0160188)

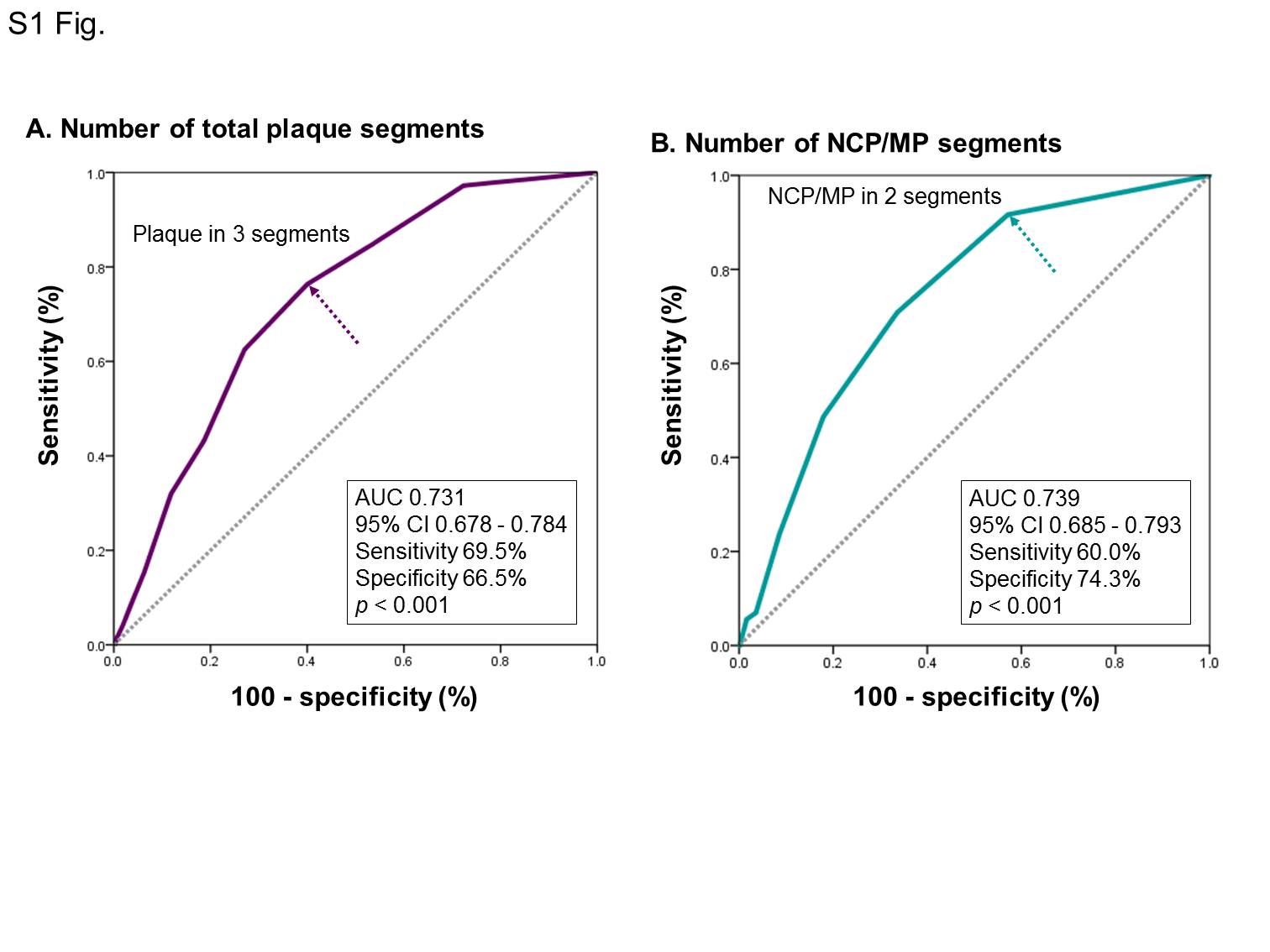

Supplement: S1 Fig — In receiver operating characteristics (ROC) analysis, the optimal cut-off for the number of involved segments is determined as 3 in the total plaque count (A) and 2 in non-calcified plaque (NCP)/mixed plaque (MP) (B), respectively. (JPG) [file pone.0160188.s001.jpg]

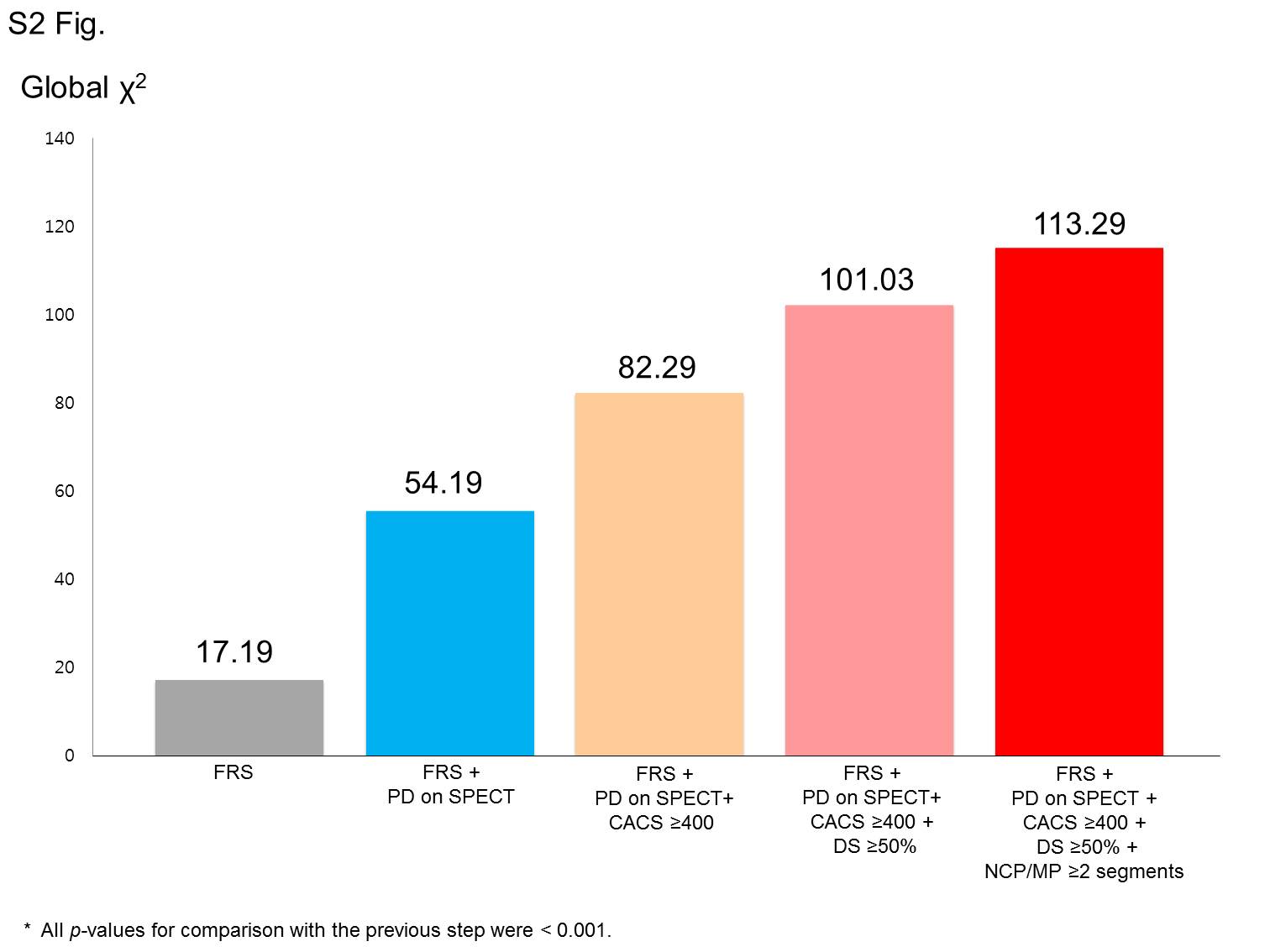

Supplement: S2 Fig — Bar graph illustrates the incremental prognostic value of cardiac CT variables in predicting adverse cardiac events by providing global χ2 scores. The addition of conventional cardiac CT variables (coronary artery calcium score [CACS] ≥400 and plaque ≥50% diameter stenosis [DS]) provides incremental prognostic information to Framingham Risk Score (FRS) and perfusion defect (PD) on single-photon emission computed tomography (SPECT). Furthermore, the addition of presence of non-calcified plaque (NCP)/mixed plaque (MP) in ≥2 segments allows further risk stratification. (JPG) [file pone.0160188.s002.jpg]

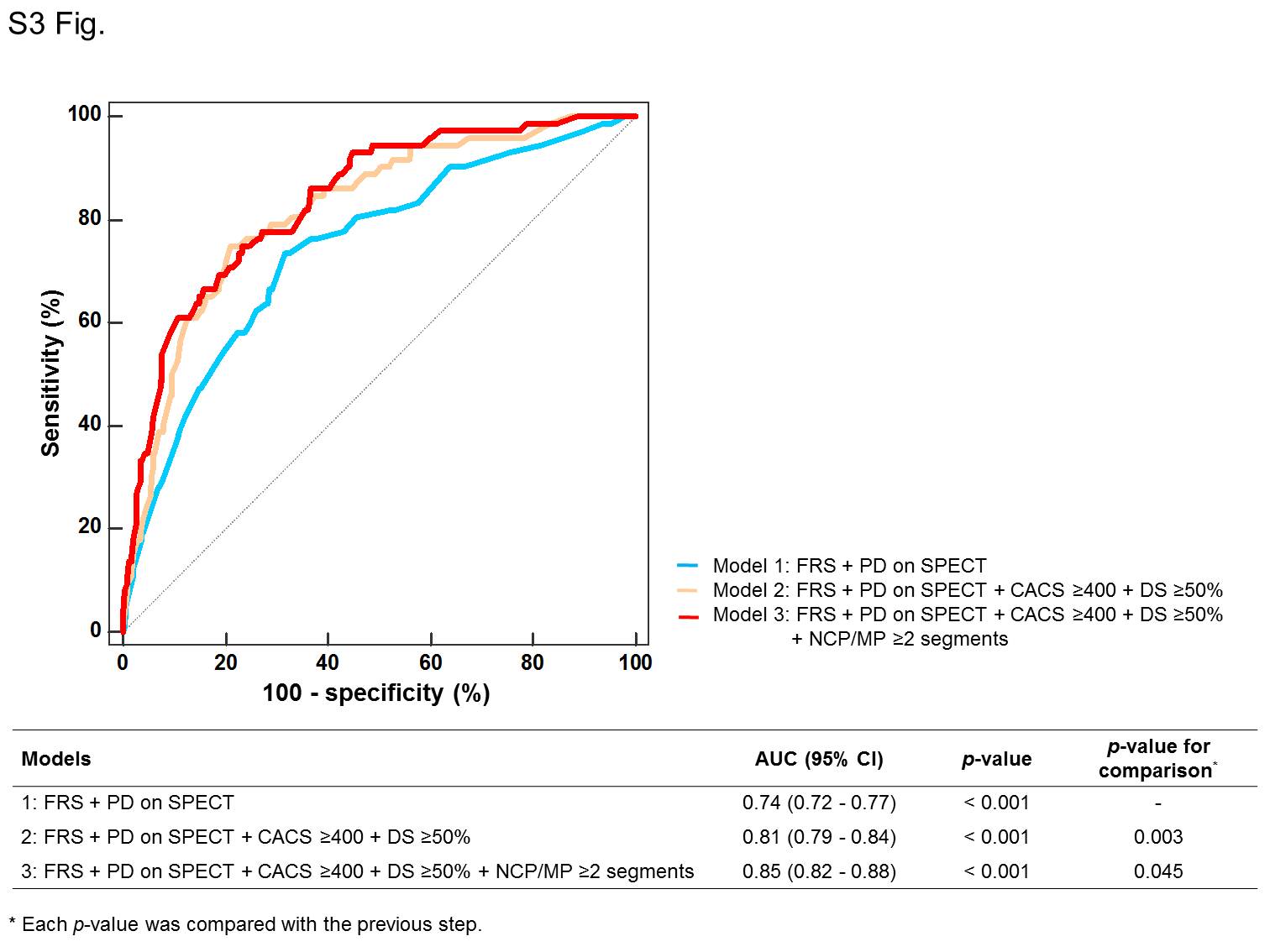

Supplement: S3 Fig — The receiver-operating characteristic (ROC) curves of 3 models depict the incremental prognostic value of cardiac CT variables in predicting adverse cardiac events by comparing AUC values. The AUC increases gradually from 0.74 (1: the Framingham Risk Score [FRS] + perfusion defect [PD] on single-photon emission computed tomography [SPECT]) to 0.81 (2: adding coronary artery calcium score [CACS] ≥400 and plaque ≥50% diameter stenosis [DS] to model 1), and from 0.81 to 0.85(3: adding non-calcified plaque [NCP]/mixed plaque [MP] in ≥2 segments to model 2). (JPG) [file pone.0160188.s003.jpg]
